# Supplementary material for: Differential Peripheral Blood Glycoprotein Profiles in Symptomatic and Asymptomatic COVID-19
Source: Viruses. 2022 Mar 7;14(3):553. doi: 10.3390/v14030553 (PMC8951729; doi:10.3390/v14030553)
Supplement: Supplementary file 1 [file viruses-14-00553-s001.zip › Table S1.pdf]

	FDR							Relative Abundance				
marker	healthy vs. sympt. COVID-19	healthy vs. sepsis	healthy vs.common cold	healthy vs. asympt. COVID-19	sympt. COVID-19 vs. sepsis	sympt. COVID-19 vs. common cold	sympt. vs.asympt.COVID-19	Sympt. COVID-19	Asympt. COVID-19	Common cold	Healthy	Sepsis
A1AT.GP001_271_5402	0.00000842	0.182	0.829	0.391	0.0062	0.000116	0.0000000151	46.53024348+I3:N42II3:M69	17.8	23.3	23.1	24.6
A1AT.GP001_271_5412	0.000263	0.0693	0.936	0.518	0.0327	0.00123	0.0000598	0.569	0.225	0.193	0.221	0.284
A1AT.GP001_271_6503	0.00715	0.102	0.0148	0.0658	0.00177	0.000264	0.00000714	0.168	0.0706	0.0718	0.107	0.0844
A1AT.GP001_271MC_5402	0.711	0.00294	0.0229	0.0306	0.0175	0.0671	0.0385	3.28	1.81	0.738	2.43	14.7
A1AT.GP001_271MC_5412	0.0000119	0.0262	0.00813	0.625	0.399	0.00000172	0.0000000659	0.172	0.074	0.0408	0.0836	0.134
A2MG.GP004_991_5402	0.0154	0.00921	0.000000509	0.0136	0.00000326	0.011	0.416	0.222	0.263	0.106	0.383	0.611
AACT.GP005_127_5401	0.66	0.00249	0.413	0.5	0.000337	0.205	0.155	0.0218	0.021	0.0199	0.0223	0.0456
AACT.GP005_127_6502	0.0581	0.000208	0.229	0.012	0.00834	0.0193	0.0000873	0.0382	0.0239	0.0272	0.0318	0.0588
AACT.GP005_271_6502	0.000919	0.0229	0.000347	0.0512	0.286	0.000153	0.00000391	0.497	0.109	0.0685	0.21	0.262
AACT.GP005_271_6503	0.196	0.000323	0.00927	0.0255	0.000112	0.000477	0.0000974	0.099	0.0598	0.0494	0.0839	0.0347
AACT.GP005_271_6513	0.000000214	0.0405	0.0141	0.474	0.00359	0.0000000193	0.0000000000845	0.0731	0.0256	0.0175	0.0293	0.0423
AGP1.GP007_93_7614	0.00000941	0.018	0.935	0.729	0.364	0.0000987	0.000000114	2.86	0.307	0.254	0.323	1.36
AGP12.GP007.008_72_6503	0.00166	0.0676	0.637	0.404	0.000362	0.00203	0.000013	9.86	4.49	4.86	5.45	3.05
AGP12.GP007.008_72_6513	0.00251	0.676	0.694	0.434	0.0175	0.0138	0.000999	0.112	0.0513	0.0443	0.0404	0.055
AGP12.GP007.008_72MC_7601	0.52	0.00317	0.838	0.508	0.00961	0.444	0.164	0.356	0.326	0.292	0.346	0.844
AGP12.GP007.008_72MC_7602	0.019	0.0000558	0.0235	0.0818	0.0439	0.000385	0.0000218	4.32	2.26	1.62	2.7	6.38
ANGT.GP009_47_5401	0.000000112	0.25	0.309	0.822	0.000751	0.00293	0.00000365	0.0472	0.127	0.097	0.18	0.125

APOB.GP013_983_5401	0.000347	0.419	0.0419	0.217	0.0439	0.0000000888	0.00853	0.0959	0.142	0.22	0.164	0.135
APOD.GP014_98_5412	0.0000237	0.00000156	0.00244	0.0878	0.00574	0.939	0.00478	0.0115	0.0166	0.0132	0.0215	0.00647
APOH.GP015_253_5401	0.000000000137	0.00699	0.00825	0.0413	0.0283	0.0143	2.7E-09	0.233	0.693	0.424	1.33	0.44
CAN3.GP022_366_6503	0.00302	0.682	0.0137	0.475	0.0443	0.0000197	0.0000209	0.0473	0.0311	0.0234	0.032	0.0334
CERU.GP023_138_5412	0.000245	0.282	0.171	0.79	0.0299	0.0349	0.00000245	0.652	0.345	0.409	0.319	0.427
CERU.GP023_762_6523	0.00128	0.542	0.368	0.57	0.028	0.017	0.00252	0.0197	0.00966	0.0126	0.0106	0.0101
CFAH.GP024_529_5402	0.0151	0.956	0.0166	0.101	0.0413	0.00000572	0.000000958	0.0225	0.0134	0.0122	0.0185	0.0178
CFAH.GP024_882_5411	0.00000566	0.124	0.00229	0.0835	0.0538	0.101	0.00929	0.00533	0.0069	0.00458	0.00758	0.0066
CFAH.GP024_882_5420.5401	0.000000000016	0.00503	0.0233	0.179	0.0139	0.00114	6.36E-11	0.00894	0.019	0.0115	0.0229	0.0128
CFAI.GP025_103_5402	0.00159	0.922	0.0139	0.022	0.0242	0.000000075	1.25E-10	0.0271	0.0171	0.0144	0.0197	0.0208
CLUS.GP026_291_5402	0.00573	0.00000112	0.000215	0.022	0.0023	0.125	0.916	0.818	0.933	0.751	1.15	0.502
CLUS.GP026_291_5421.5402	0.000245	0.000000332	0.000564	0.0183	0.0103	0.881	0.239	0.465	0.657	0.526	0.789	0.264
CLUS.GP026_86_6503	0.0000436	0.000000479	0.124	0.0141	0.00378	0.0865	0.196	0.0113	0.0151	0.0147	0.02	0.00431
CO2.GP027_621_6301	0.611	0.000713	0.137	0.0616	0.00898	0.108	0.0242	0.0444	0.021	0.0206	0.0345	0.116
CO8B.GP034_243_6610	0.0000000964	0.000000332	0.0585	0.00192	0.0359	0.0158	0.177	0.716	1.06	1.23	2.07	0.334
FETUA.GP036_346_1101	0.000000189	0.00000194	0.0025	0.146	0.02	0.373	0.0000187	5.92	9.82	6.93	10.2	3.2
FETUA.GP036_346_1102	0.000467	0.00000197	0.00365	0.172	0.0188	0.924	0.0632	0.371	0.481	0.426	0.574	0.225
HEMO.GP042_240.246_5402	0.000877	0.00000112	0.00486	0.0972	0.00032	0.428	0.246	0.0303	0.034	0.0269	0.0407	0.018
HEMO.GP042_64_5401	2.07E-12	0.0418	0.0298	0.57	0.00861	0.0000639	2.77E-12	0.0373	0.0651	0.0526	0.0753	0.0518

HPT.GP044_184_6411	2.96E-08	0.0146	0.144	0.813	0.00032	0.0000000618	2.81E-10	1.04	0.312	0.14	0.31	0.536
HPT.GP044_241_5411	0.000205	0.932	0.112	0.00592	0.00177	0.13	0.871	0.03	0.0325	0.0412	0.0591	0.0614
HPT.GP044_241_7602	0.0367	0.000207	0.215	0.367	0.0363	0.00503	0.000634	1.09	0.665	0.601	0.661	1.66
HRG.GP045_125_5420.5401	1.35E-16	0.0000334	0.0727	0.0165	0.0557	0.00000000043	8.72E-10	0.0736	0.206	0.237	0.285	0.111
IC1.GP077_253_5412	0.000276	0.197	0.00379	0.0165	0.0252	0.00000172	2.84E-09	0.152	0.065	0.0534	0.0912	0.114
IC1.GP077_253_6513	0.00789	0.203	0.000555	0.000788	0.00217	0.00000172	2.32E-09	0.0614	0.0299	0.0299	0.0448	0.0391
IGG1.GP048_297_3410	0.0266	0.00519	0.0506	0.721	0.0413	0.00375	0.00293	27	14.8	11.1	15.7	36
IGG1.GP048_297_3510	0.346	0.0343	0.00878	0.891	0.0432	0.00892	0.212	4.94	3.72	2.53	4.13	6.51
IGG2.GP049_297_4310	0.000586	0.0000513	0.0193	0.0151	0.0357	0.911	0.912	0.0387	0.0391	0.0419	0.0651	0.0268
IGG2.GP049_297_4400	0.000378	0.00000543	0.00472	0.0357	0.0359	0.826	0.421	0.141	0.2	0.139	0.305	0.0776
IGG2.GP049_297_4410	0.000000913	0.00000364	0.000944	0.00864	0.0175	0.533	0.278	8.89	11.2	8.61	21.1	4.41
IGG2.GP049_297_4411	0.000000194	0.00000112	0.000419	0.00246	0.0246	0.569	0.407	1.98	2.15	1.51	4.12	0.861
IGG2.GP049_297_4511	0.0000000313	0.00000000728	0.00109	0.00147	0.0269	0.475	0.27	0.00595	0.00754	0.00675	0.0155	0.00302
IGG2.GP049_297_5410	0.00000000000553	0.000000332	0.00176	0.00246	0.0107	0.023	0.00133	3.92	7.53	6.09	17.2	1.34
IGG2.GP049_297_5411	0.00000000194	0.000000571	0.0117	0.00166	0.0258	0.057	0.0858	2.02	3.03	3.13	6.78	0.769
IGG2.GP049_297_5510	0.000000000363	0.000000363	0.0126	0.0168	0.0297	0.0184	0.00249	0.18	0.38	0.372	0.87	0.116
ITIH1.GP054_285_5402	0.00000864	0.0145	0.695	0.559	0.0331	0.000067	0.000000507	0.0227	0.00558	0.0052	0.00576	0.0118
ITIH1.GP054_285_5402.5421	0.00000000763	0.00000501	0.76	0.079	0.0235	0.0000000798	0.0000471	0.045	0.0599	0.0739	0.07	0.0365
ITIH1.GP054_285_5511	0.0000000585	0.0000095	0.00676	0.0107	0.0139	0.713	0.405	0.00948	0.0101	0.00936	0.0148	0.00753

ITIH1.GP054_588_5402	0.291	0.000624	0.000141	0.578	0.00995	0.0000241	0.7	0.00834	0.00807	0.00407	0.00937	0.00543
ITIH1.GP054_653_1101	0.00000000167	0.00000202	0.047	0.058	0.0107	0.000138	0.00000311	0.371	0.571	0.549	0.658	0.28
KLKB1.GP056_396_5402	0.000593	0.000995	0.418	0.0962	0.0413	0.0217	0.279	0.175	0.187	0.22	0.216	0.134
KLKB1.GP056_453_5402	0.00158	0.000512	0.295	0.248	0.0323	0.139	0.0844	0.134	0.154	0.157	0.179	0.101
KLKB1.GP056_494_5401	0.815	0.0483	0.204	0.204	0.0175	0.269	0.231	0.00117	0.00103	0.00112	0.00124	0.0026
KNG1.GP057_628_1102	0.0000105	0.0000284	0.0125	0.0263	0.0175	0.826	0.4	0.291	0.291	0.27	0.417	0.184
QUANTPEP.A2GL.GP003_DLLLPQPDLR	0.000000378	0.000000782	0.0286	0.0427	0.00861	0.000000351	0.000000000053	3.9	1.1	1.01	1.42	6.46
QUANTPEP.ANT3.GP010_FATTFYQHLADSK	0.0000000000102	0.00000141	0.00264	0.000212	0.515	0.00175	0.0845	0.0556	0.066	0.08	0.126	0.0515
QUANTPEP.APOA1.GP011_DLATVYVDVLK	0.0000131	0.000000806	0.422	0.696	0.000883	0.000951	0.00000125	24.8	47.6	43	49	12
QUANTPEP.CLUS.GP026_ASSIIDELFQDR	0.0061	0.000000575	0.000149	0.0178	0.00154	0.0839	0.99	1.56	1.71	1.34	2.09	0.964
QUANTPEP.KNG1.GP057_YFIDFVAR	0.0000000126	0.00000288	0.0000879	0.0456	0.0269	0.0191	0.00106	5.59	7.64	4.88	8.87	4.5
QUANTPEP.PON1.GP060_YVYIAELLAHK	0.000000883	0.00461	0.583	0.578	0.919	0.0000356	0.000000232	0.241	0.378	0.352	0.355	0.251
QUANTPEP.SEPP1.GP061_VSLATVDK	0.00000931	0.000000332	0.021	0.0881	0.00133	0.112	0.002	0.105	0.14	0.135	0.188	0.0627
QUANTPEP.TTR.GP065_TSESGELHGLTTEEEFVEGIYK	0.000291	0.00000014	0.00043	0.0205	0.000000000756	0.00145	0.00000000000000533	0.0338	0.0694	0.0206	0.0459	0.00709
THRB.GP063_143_5402	0.0819	0.0000946	0.76	0.0788	0.00574	0.0389	0.702	3.4	3.27	4.14	3.97	2.27

THRB.GP063_416MC_5401	0.0000000000795	0.124	0.00201	0.179	0.0000134	0.0541	0.00000000248	0.0393	0.102	0.0569	0.121	0.0886
UN13A.GP066_1005_7420	0.00000302	0.0000154	0.0594	0.515	0.025	0.0258	0.00000205	0.0647	0.124	0.0938	0.136	0.0327
VTNC.GP067_169_5301	0.00693	0.00105	0.838	0.407	0.0127	0.0281	0.000109	0.722	0.394	0.483	0.47	1.17
VTNC.GP067_86_5421	0.145	0.00636	0.00109	0.746	0.0163	0.209	0.0427	0.814	0.641	1.28	0.641	0.47
ZA2G.GP068_112_5420	0.000000866	0.686	0.232	0.578	0.0327	0.00101	0.000000392	0.00921	0.0195	0.0153	0.0238	0.0135
