# Supplementary material for: Differential Peripheral Blood Glycoprotein Profiles in Symptomatic and Asymptomatic COVID-19
Source: Viruses. 2022 Mar 7;14(3):553. doi: 10.3390/v14030553 (PMC8951729; doi:10.3390/v14030553)
Supplement: Supplementary file 1 [file viruses-14-00553-s001.zip › Table S2.pdf]

viruses

MDPI

Supplementary Materials

Differential Peripheral Blood Glycoprotein Profiles in Symptomatic and Asymptomatic COVID-19

Table S2. Detailed description of glycoprotein biomarkers used in LASSO-regularized classifier.

Biomarker	Protein	Glycosylation site	Glycan structure	Glycan figure
**A1AT.GP001_271_6503**	Alpha-1-antitrypsin	271	6503	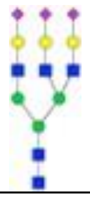
**A1AT.GP001_271MC_5412**	Alpha-1-antitrypsin	271MC	5412	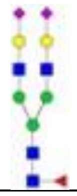
**A2MG.GP004_991_5402**	Alpha-2-macroglobulin	991	5402	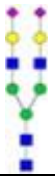
**AACT.GP005_271_6502**	Alpha-1-antichymotrypsin	271	6502	
**AACT.GP005_271_6503**	Alpha-1-antichymotrypsin	271	6503	
**AGP1.GP007_93_7614**	Alpha-1-acid glycoprotein 1	93	7614	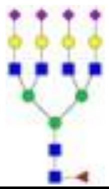
**ANGT.GP009_47_5401**	Angiotensinogen	47	5401	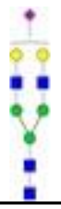

**CFAH.GP024_882_5411**	Complement Factor H	882	5411	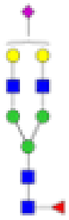
**CFAH.GP024_882_5420.5401**	Complement Factor H	882	5401	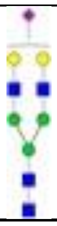
**HEMO.GP042_64_5401**	Hemopexin	64	5401	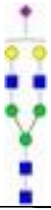
**HPT.GP044_184_6411**	Haptoglobin	184	6411	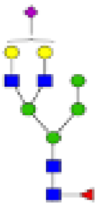
**HPT.GP044_241_5411**	Haptoglobin	241	5411	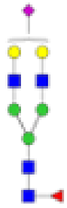
**HRG.GP045_125_5420.5401**	Histidine-rich glycoprotein	125	5401	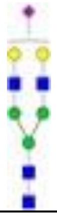
**IC1.GP077_253_6513**	Plasma protease C1 inhibitor	253	6513	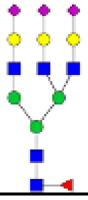

**ITIH1.GP054_285_5402**	Inter-alpha-trypsin inhibitor heavy chain H1	285	5402	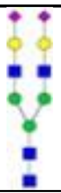
**QUANTPEP.ANT3.GP010_FATTFYQHLADSK**	Antithrombin-III	PEP	FATTFYQHLADSK	N/A
**QUANTPEP.PON1.GP060_YVYIAELLAHK**	Serum paraoxonase/arylesterase 1	PEP	YVYIAELLAHK	N/A
**THRB.GP063_416MC_5401**	Prothrombin	416MC	5401	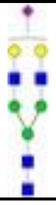
